# Marijuana Content on Digital Media and Marijuana Use among Young People in the United States

**DOI:** 10.26828/cannabis/2022.02.007

**Published:** 2022-07-11

**Authors:** Yoonsang Kim, Lisa Vera, Jidong Huang, Sherry Emery

**Affiliations:** 1 Social Data Collaboratory, Department of Public Health, NORC at the University of Chicago; 2 VeraCite Inc.; 3 Department of Health Policy & Behavioral Sciences, School of Public Health, Georgia State University

**Keywords:** Twitter, Google Trends, marijuana use, public communication environment

## Abstract

Health behavior theory establishes that exposure to media messages about a topic influences related knowledge, attitudes, and behavior. Marijuana-related messages proliferating on digital media likely affect attitudes and behavior about marijuana. Most research studying marijuana-related media effects on behavior relies on self-reported survey measures, which are subject to bias; people find it difficult to recall timing, frequency, and sources of messages. We calculated an exogenous measure of exposure to marijuana-related messages on digital media based on emerging public communication environment (PCE) theory. Aggregated online searches and social media posts related to marijuana for a given place reflect the marijuana-related PCE, where people are exposed to and engage with messages from multiple sources. Exogenous measures overcome bias in self-reported exposure and outcome data: simultaneity bias and endogeneity. The PCE reflects both potential exposure and relative importance of the topic in the local community, which may influence real-world marijuana use. Using 2017 Twitter and Google Search data, we measured the marijuana-related PCE to quantify where opportunities for exposure to marijuana-related posts were high and examined relationships between potential exposure and current marijuana use among youth and young adults in 2018. We found that marijuana-related online search and tweeting at the media market level are associated with offline marijuana use, controlling for demographics and state marijuana policy. The marijuana-related digital media environment may reflect and/or influence youth and young adult marijuana use. Social media and online search data offer platforms to monitor the marijuana-related PCE and supplement survey data to study media exposure and marijuana use behavior.

Multiple health behavior models have documented the finding that exposure to messages about a topic influences knowledge, attitudes, and behavior related to that topic (Ajzen & Fishbein, 1980; Fishbein & Ajzen, 1975; McGuire, 1985; Pierce et al., 2017). Digital media (e.g., social media, websites, online ads) are an important channel for marijuana-related marketing, consumer engagement, and policy advocacy (Clayton, 2021; Leafly, 2015). Retailers advertise cannabis products on social media, often using tactics appealing to young people – e.g., celebrity/influencer endorsement and promotion (Bierut et al., 2017; Lim et al., 2021). Social media posts, such as on the popular site Twitter, also reflect attitudes toward marijuana policy, with more marijuana-related communications with positive sentiment generated in states with legal recreational marijuana policies (Daniulaityte et al., 2017; van Draanen et al., 2020).

Young people may learn and reinforce their behaviors by observing others through digital media. Young people are exposed to and search for cannabis-related information and express intentions, opinions, and beliefs on digital media (Cavazos-Rehg et al., 2014; Yang et al., 2018). Many such messages convey positive sentiment and normalize marijuana use (Cavazos-Rehg, Krauss, et al., 2016; Cavazos-Rehg, Sowles, et al., 2016), with potential influence on attitudes, beliefs, and behaviors. For example, @*E***J*** posted a tweet saying “Beat the heat and stay chill this summer with these ultra dank weed ice creams” with a link to its website marketing cannabis ice cream.

Emerging research has studied the association between marijuana-related digital media and marijuana use. One study found young adult marijuana use to be associated with active and passive exposure to marijuana-related tweets (Cabrera-Nguyen et al., 2016). A study with 18–34-year-old past-month marijuana users found that over half viewed marijuana ads in the previous month, commonly on digital media; those who actively sought ads often used Internet search engines and social media, and ad exposure was associated with heavier use (Krauss et al., 2017). Further research discovered that cannabis advertising exposure differed by policy jurisdiction, social media was a frequently-cited advertising channel, and higher ad exposure was associated with higher marijuana use rates (Rup et al., 2020). However, these studies measure exposure and outcomes via survey, likely introducing both simultaneity bias and endogeneity. In the highly-fragmented digital environment, self-reported exposure has bias because people have difficulty accurately recalling where, when, and how often they saw and engaged with messages (Slater, 2004). An alternative approach to measuring exposure is needed to avoid such bias.

An exogenous measure offers an alternative to surveys for assessing potential exposure, and communication theory provides a framework for such a measure (Liu & Hornik, 2016). People are exposed to and engage with messages from multiple communication sources, which constitute the public communication environment (PCE) (Hornik et al., 2019; Hornik et al., 2022). Similar to using television ratings as an objective measure of how many people see a televised program or advertisement (Emery et al., 2012; Layton et al., 2017), we can consider the aggregation of online searches and social media posts on a given topic and place to reflect the local PCE. Because individuals’ online social networks overlap substantially with their offline social networks (Dunbar et al., 2015), we can measure the local marijuana-related PCE by aggregating geolocated messages about marijuana. This exogenous measure of potential exposure may enable inferences about how those messages affect individual behavior.

The local PCE reflects both opportunity for exposure and relative importance of the topic in the local community (Hornik et al., 2022). For example, in communities where retailers post social media messages about products promotions and marketing, local enthusiasts may follow the shops’ accounts and thus have direct message exposure; they may also re-post or share the messages, increasing potential exposure among their followers (Cygnis Media Editor, 2012; Fishbein & Hornik, 2008; Hothi, 2012). Similarly, individuals may deliberately seek information about the topic using online search engines; this sought information also reflects the PCE. Online search increases chances of exposure to relevant information, and greater relative search frequency by a given community implies higher interest in the topic. Hornik et al. (Hornik et al., 2013) differentiate between information-*seeking* and information-*scanning* behaviors, positing that while a single exposure to a topic as a result of a deliberate search may be more influential than a single episode of scanning behavior on the same topic, scanning behavior is much more frequent and – when taken in the aggregate – may be more influential. Proliferation of messages reflects and influences community norms, culture, and marketing that constitute the local marijuana-related PCE, which may influence real-world marijuana use.

Using Twitter and Google Search data, we can measure the local PCE related to marijuana and examine where and when opportunities for exposure to marijuana-related content were high. Google is currently the top search engine with over 80% market share. As of 2018 32% of online teens used Twitter (Anderson & Jiang, 2018; Smith & Anderson, 2018), and as of 2021, 42% of young adults (aged 18–29) used Twitter (Auxier & Anderson, 2021). Both Google Search and Twitter have been considered important data sources to monitor and address emerging public health and epidemiological issues (Eichstaedt et al., 2015; Ginsberg et al., 2009; Jordan et al., 2019; Pelat et al., 2009; Seifter et al., 2010). This paper explores whether self-reported marijuana use among US youth and young adults is associated with marijuana-related Google Search volume and tweets.

## METHODS

### Data and Measures

A US representative sample of 6,684 youth and adults were interviewed about their tobacco use, marijuana use, and sociodemographic characteristics between April-June 2018. We selected a sample of youth and young adults (13–24 years) for this study. A general population sample of youth (13–17 years) and young adults (18–24 years) was selected from NORC’s AmeriSpeak Panel and GfK’s Knowledge Panel for this study. A total of 3,886 respondents completed the survey (1,810 from AmeriSpeak Panel and 2,076 from KnowledgePanel). Of those, 43 did not report on marijuana use and 1 respondent did not provide information about where they lived; the final sample size was 3,842. The survey was offered in English, via phone or online for the AmeriSpeak Panel and online only for the Knowledge Panel. Cumulative response rate was 4.5% for KnowledgePanel teens, 7.0% for AmeriSpeak teens, 3.5% for KnowledgePanel adults, and 9.2% for AmeriSpeak adults; cumulative response rate for the sample of youth and young adults was not separately calculated. Statistical weighting was performed to account for nonresponse, subgroup oversampling, and combining two panels in reference to population total benchmarks for age, race/ethnicity, education, gender, census division, and e-cigarette ever-use. Respondents were asked “Do you now use marijuana or hashish?” and categorized as current marijuana users if they reported “every day” or “some days” use.

Google search volume data (March 2017–February 2018) were compiled by Designated Market Area (DMA) from Google Trends (http://trends.google.com), using keywords ‘marijuana’ and ‘cannabis.’ Google Trends data represent relative popularity of the search term; relative search volume (RSV) is defined as the number of searches for a particular term relative to the total number of searches done on Google during the observation period. The RSV is then divided by the highest number of searches for the particular term during the observation period, resulting in a value that ranges from 0–100 (Google News Initiative, n.d.). Duplicate searches done by the same person are excluded. DMAs are comprised of contiguous counties typically centered in and near large cities and correlated with metropolitan areas.

Marijuana-related tweets (January–December 2017) were collected from Gnip’s Historical PowerTrack using marijuana-related search queries. Our search queries included prominent accounts that marketed marijuana products or advocated marijuana use and relevant policy change; in addition, our queries included terms indicating marijuana product, use, and regulation. We developed the queries using Boolean operators for a focused search; for instance, “smoke” AND “kush”; “smoking” AND “legalize” NOT “cigarette.” Our general strategy to develop and test search queries is described elsewhere (Kim et al., 2016). The complete list of search queries is available upon request. About 3.17 million marijuana-related tweets were collected, of which 952,428 (30%) were geolocated to DMAs. We used two pieces of location data provided by Twitter: user-tagged locations and Gnip’s predicted locations. The latter is based on information extracted from user profiles; many users publicly indicate location either by selecting a city and state from a preset list or by directly typing place names in their profiles. Gnip uses this information to geolocate Twitter users’ locations by matching place names against the GeoNames.org database. More details for identifying tweet geolocation and the *fitness for use* of geolocated tweets are reported elsewhere (Kim et al., 2020). Tweet sentiment was assessed using VADER, resulting in a score between −100 (highly negative) and 100 (highly positive) (Hutto & Gilbert, 2014). VADER calculates standardized sentiment score based on lexicon ratings (degree of positive, neutral, and negative) and the proportion of the text that falls into each sentiment category. We calculated an average sentiment score over tweets posted from each DMA.

### Statistical Analysis

Average sentiment score of tweets and Google search volumes were linked with the survey data based on DMAs where respondents lived. The bivariate relationships between marijuana use and Google search volume as well as tweet sentiment score were analyzed by age group (youth and young adults). We categorized Google search volume and tweet sentiment score by quartile and calculated the prevalence of current marijuana use and 95% confidence interval within each category. We used multivariate logistic models to control for individual-level covariates likely associated with marijuana use, including age group, sex, race/ethnicity, household income, and state marijuana policy in 2018 (legal adult use vs. other). In 2018, recreational marijuana use was legal in nine states and the District of Columbia. However, the legalization law in Vermont took effect on July 2018, which was after the survey was in the field; thus, we treated Vermont as one of the “other” states. Three models were estimated to understand whether and to what extent digital media measures additionally explain variation in the outcome: model without digital media measures; model with search volume; model with tweet sentiment score. The effects of digital media and marijuana policy on marijuana use may differ by age. Thus, the models also included interactions of digital media and age group as well as marijuana policy and age group; we calculated odds ratios for digital media measures and marijuana policy for youth and young adults separately. Weighted prevalence and odds ratios were estimated using SAS/STAT version 15.1. Volumes of ‘marijuana’ search and ‘cannabis’ search on Google showed very similar relationships with current marijuana use. We present the results based on ‘marijuana’ search volume.

## RESULTS

Summary statistics of demographics and state marijuana policy for youth and young adult respondents by marijuana use status are presented in Table 1. Of marijuana users, 30.4% had household income <$25,000, while 20.9% of non-marijuana users had income <$25,000. Marijuana users were more likely to be Black and Latino than non-users. As anticipated, larger proportions of marijuana users lived in states with legal adult marijuana use (29.7% vs. 19.9%).

**Figure 1.**
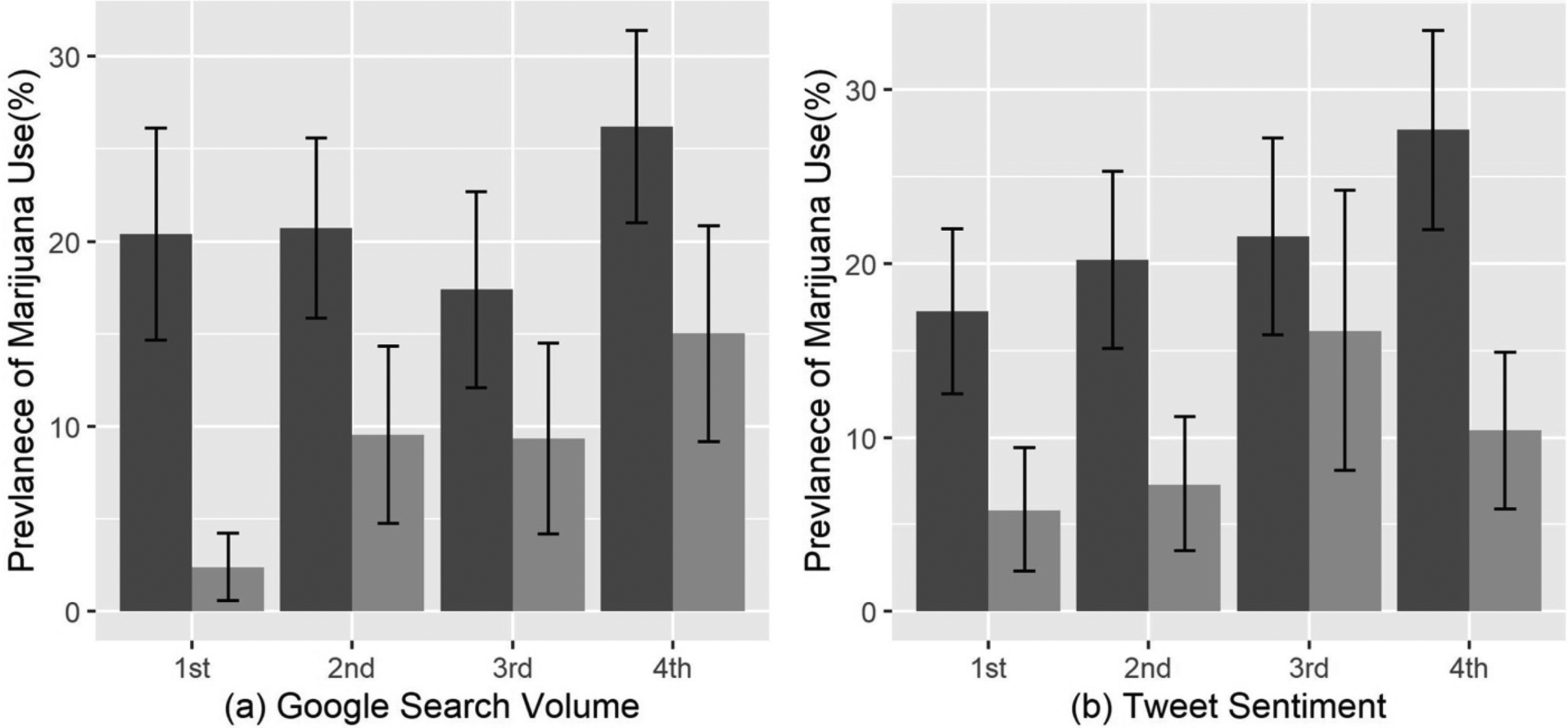
Current Marijuana Use, Marijuana Search Volume on Google Trends, and Sentiment of Marijuana-Related Tweets *Note*. Prevalence for young adults is indicated by dark grey bars █ and prevalence for youth is indicated by light grey bars █. Current marijuana use and cannabis search volume showed very similar trend displayed in (a).

Prevalence of current marijuana use was 9.7% (CI=7.2%, 12.3%) among youth and 21.7% (CI=19.0%, 24.3%) among young adults. Figure 1 displays bivariate relationships relationships between current marijuana use and (a) ‘marijuana’ search volume on Google and (b) sentiment scores of marijuana-related tweets. Overall, both digital media measures exhibit positive relationships with marijuana use. Youth living in the DMAs in the highest quartile of marijuana search volume were more likely to report current marijuana use than those living in DMAs in the lowest quartile (15.0% [CI=9.2%, 20.9%] vs. 2.4% [CI=0.6%, 4.2%]). Prevalence of young adults’ marijuana use appears to increase with the tweet sentiment.

Table 2 presents three multivariate logistic regressions: (1) model without digital media, (2) model with search volume, and (3) model with tweet sentiment. Age group was significantly associated with reporting current marijuana use; the odds of marijuana use for young adults were larger than twice that for youth, controlling for other covariates. Latino and non-Latino Black respondents were more likely to use marijuana than non-Latino White respondents. In the first model without digital media measures included, state marijuana policy was strongly associated with current marijuana use; those living in states where recreational use was legal were more likely to use marijuana. The second model with Google Search volume showed that for youth, the odds of reporting current marijuana use was 79% greater (OR=1.79; CI=1.23, 2.60) as the search volume increased by one standard deviation (=13.8), while a similar pattern was not observed for young adults. The third model with tweet sentiment score showed that for young adults, the odds of reporting current marijuana use was 27% greater (OR=1.27; CI=1.01, 1.59) as tweet sentiment score changed by one standard deviation (=5.4) in a positive direction, although a similar pattern was not observed for youth. Interestingly, the association of state marijuana policy with the outcome was dampened when digital media measures were included.

State policy was associated with the outcome as well as both digital media measures. Youth and young adults living in states where recreational marijuana use was legal had higher search volume on average (47.9% vs. 27.3%). Similarly, states with recreational laws had tweets with an average sentiment score indicating neutral sentiment, while states without recreational laws had tweets with slightly more negative sentiment (−0.001 vs. −0.041).

## DISCUSSION

This study sought to determine associations between marijuana-related tweets and Google search volume and self-reported marijuana use among youth and young adults in the US. We found that DMA-level marijuana-related online search was associated with youth marijuana use and tweeting was associated with young adult marijuana use, controlling for demographics and state marijuana policy. Our findings suggest that the marijuana-related digital media environment may reflect and possibly influence marijuana use among youth and young adults. Further research is required to understand why different types of digital media are associated with offline marijuana use by age group.

Previous studies reported that youth and young adult marijuana use was associated with *self-reported* exposure to marijuana advertisements on the Internet (Dai, 2017; Krauss et al., 2017; Rup et al., 2020) and marijuana-related tweets (Cabrera-Nguyen et al., 2016). Our study demonstrates that community-level digital media data can provide valuable and rapid measures of potential exposure to marijuana-related information on digital media. These exogenous measures reflect the community environment and social norms surrounding marijuana use and overcome bias in self-reported exposure data; and can be used as an indicator of the likelihood that an individual residing in a region may encounter marijuana-related messaging. Thus, digital media data may be used as a proxy for the local PCE around marijuana use and supplement survey data to enhance understanding of factors influencing young people’s substance use.

We also observed that state marijuana policy was associated with marijuana-related tweets and Google Search; this correlation suggests that the PCE may either reflect and/or influence the policy environment, and both appear to be associated with marijuana use. We considered DMA as the unit of PCE measures to quantify opportunities for exposure to marijuana-related digital media content. DMA has been widely used as the unit of measure to analyze the media effect. Twitter users may indicate the core city as their location, although they may reside in a suburban area, and people easily travel within metropolitan areas. Most DMAs are defined based on metropolitan areas and often cross state boundaries. On the other hand, the legality of marijuana use is bounded by state and associated with relevant attitudes and use of state residents. However, people may purchase marijuana in neighboring states. In 2018, recreational marijuana use was legal in nine states and the District of Columbia, although the sale and purchase remain illegal in the District of Columbia.

Some study limitations must be stated. We used Twitter and Google search data, whereas several more media channels contribute to the PCE. We did not consider legacy media sources, such as The New York Times, which also comprise part of the PCE. However, we believe our measures still reflect the PCE, and much legacy media content can be found on Twitter (e.g., @nytimes) and via Google Search. Second, the level of individuals’ exposure may vary from the exogenous measure. Using an exogenous measure of exposure to topical messaging may be complicated by the fact that consumers’ digital media patterns are recorded in cookies and search histories and used to fine-tune targeted marketing strategies. Nonetheless, our team has demonstrated in prior work that such measures are correlated with tobacco product sales and youth attitudes and beliefs about tobacco use (Berg et al., 2019; Liu et al., 2019). Third, using the VADER to calculate sentiment score may not accurately capture pro- versus anti-marijuana opinions; it reflects emotional valence in tweets, and some pro-marijuana tweets may convey negative sentiment, for example. Finally, we analyzed the survey data collected in late spring 2018, linking with 2018 state marijuana policy, tweets posted in January–December 2017, and Google Search data from March 2017–February 2018. Since then, more states have legalized recreational marijuana use, and digital media related to marijuana have likely evolved. However, we believe that our findings still speak to the relationship between marijuana-related PCE and marijuana use.

Our findings suggest that youth and young adults living in areas where more people search for cannabis information on Google and post cannabis-related tweets with positive sentiment are more likely to use marijuana. Social media and online search data may offer platforms to monitor the cannabis-related PCE and supplement survey data to study media exposure and substance use behavior.

## Figures and Tables

**Table 1.** Summary Statistics for Sociodemographics of Youth and Young Adults by Marijuana Use Status

Variables		Marijuana users^a^			Marijuana non-users		
		N	Mean/%	(95% CI)	N	Mean/%	(95% CI)
Age	Youth	101	23.9	(18.1, 29.7)	1932	44.6	(42.2, 47.1)
	Young adult	402	76.1	(70.3, 81.9)	1408	55.4	(52.0, 57.8)
Gender	Female	290	47.6	(41.4, 53.8)	1984	52.5	(49.9, 55.0)
	Male	213	52.3	(46.1, 58.5)	1355	47.5	(44.9, 50.0)
Income	<$25,000	196	30.4	(25.1, 35.6)	732	20.9	(18.9, 23.0)
	$25,000–49,999	121	20.2	(15.7, 24.7)	729	22.2	(20.0, 24.4)
	$50,000–99,999	113	27.3	(21.4, 33.3)	1095	31.2	(28.9, 33.5)
	$100,000+	72	22.1	(16.3, 28.0)	783	25.6	(23.5, 27.8)
Race/Ethnicity	White NH^b^	164	44.4	(38.2, 50.7)	1726	56.1	(53.6, 58.5)
	Black NH^b^	132	17.9	(13.9, 21.8)	519	12.9	(11.3, 14.4)
	Other NH^b^	8	1.4	(0.2, 2.7)	62	1.9	(1.3, 2.6)
	Hispanic	134	26.9	(21.0, 32.8)	674	21.8	(19.7, 23.8)
	Multirace NH^b^	40	5.7	(3.1, 8.3)	167	4.0	(3.1, 5.0)
	Asian NH^b^	25	3.6	(0.9, 6.2)	191	3.3	(2.5, 4.1)
Policy^c^	Other	375	70.3	(64.5, 76.0)	2769	80.1	(78.0, 82.2)
	Legal	128	29.7	(24.0, 35.4)	570	19.9	(17.8, 22.0)

a Current marijuana use was measured by asking “Do you now use marijuana or hashish every day, some days, or not at all?” Every day and some days users were categorized as current marijuana users.

b NH: non-Hispanic/non-Latinx

c State marijuana policy: legal for adults/recreational vs. other (reference)

**Table 2.** Multivariate Logistic Models of Current Marijuana Use^a^ and Digital Media

Variables		No digital media			Marijuana search			Tweet sentiment		
		OR	(95% CI)	p	OR	(95% CI)	p	OR	(95% CI)	p
Age^b^	Youth	1.00		<.001	1.00		<.001	1.00		<.001
	Young adult	2.83	(1.90, 4.21)		2.59	(1.71, 3.93)		2.87	(1.92, 4.28)	
**Youth**										
Digital media^c^			--		1.79	(1.23, 2.60)	.002	1.19	(0.91, 1.55)	.200
Policy^d^	Other	1.00		.022	1.00		.906	1.00		.060
	Legal	2.16	(1.12, 4.15)		0.95	(0.41, 2.22)		1.93	(0.97, 3.84)	
**Young adult**										
Digital Media^b^			--		1.19	(0.88, 1.61)	.254	1.27	(1.01, 1.59)	.037
Policy^d^	Other	1.00		.038	1.00		.688	1.00		.308
	Legal	1.48	(1.02, 2.15)		1.12	(0.63, 1.99)		1.23	(0.82, 1.84)	
**Youth & young adult**										
Gender	Female	1.00		.232	1.00		.240	1.00		.279
	Male	1.18	(0.90, 1.57)		1.18	(0.89, 1.57)		1.17	(0.88, 1.54)	
Income	<$25,000	1.00		.234	1.00		.190	1.00		.259
	$25,000–49,999	0.69	(0.49, 0.98)		0.68	(0.47, 0.97)		0.70	(0.49, 0.99)	
	$50,000–99,999	0.83	(0.58, 1.19)		0.83	(0.58, 1.18)		0.83	(0.58, 1.18)	
	$100,000+	0.86	(0.57, 1.32)		0.90	(0.59, 1.37)		0.87	(0.57, 1.32)	
Race/Ethnicity	White NH^e^	1.00		.023	1.00		.008	1.00		.013
	Black NH^e^	1.76	(1.24, 2.50)		1.89	(1.33, 2.69)		1.84	(1.29, 2.61)	
	Other NH^e^	0.70	(0.28, 1.77)		0.74	(0.29, 1.86)		0.73	(0.29, 1.85)	
	Hispanic	1.47	(0.99, 2.17)		1.57	(1.06, 2.32)		1.51	(1.02, 2.24)	
	Multirace NH^e^	1.66	(0.92, 2.99)		1.65	(0.90, 3.00)		1.72	(0.95, 3.11)	
	Asian NH^e^	1.32	(0.58, 3.03)		1.36	(0.60, 3.08)		1.33	(0.58, 3.07)	

a Current marijuana use was measured by asking “Do you now use marijuana or hashish every day, some days, or not at all?” Every day and some days users were categorized as current marijuana users.

b The odds ratios for young adults vs. youth were calculated conditional on the mean values of digital media measures and for those living in states where marijuana use is not legal for adults. The odds ratios conditional on legal marijuana use are greater.

c Marijuana search volume (March 2017–February 2018) and tweet sentiment scores (January–December 2017) were divided by their standard deviations to estimate the odds ratio for one standard deviation change.

d State marijuana policy: legal for adults/recreational vs. other (reference)

e NH: non-Hispanic/non-Latinx
